# MR-orthopantomography in operative dentistry and oral and maxillofacial surgery: a proof of concept study

**DOI:** 10.1038/s41598-023-33483-7

**Published:** 2023-04-17

**Authors:** Adib Al-Haj Husain, Valérie Schmidt, Silvio Valdec, Bernd Stadlinger, Sebastian Winklhofer, Daphne Schönegg, Stefan Sommer, Mutlu Özcan, Nadin Al-Haj Husain, Marco Piccirelli

**Affiliations:** 1 Clinic of Cranio-Maxillofacial and Oral Surgery, Center of Dental Medicine, University of Zurich, Plattenstrasse 11, CH-8032 Zurich, Switzerland; 2 Department of Neuroradiology, Clinical Neuroscience Center, University Hospital Zurich, University of Zurich, Zurich, Switzerland; 3 Departement of Cranio-Maxillo-Facial and Oral Surgery, University Hospital Zurich, Zurich, Switzerland; 4 Siemens Healthineers International AG, Zurich, Switzerland; 5 Swiss Center for Musculoskeletal Imaging (SCMI), Balgrist Campus, Zurich, Switzerland; 6 Advanced Clinical Imaging Technology (ACIT), Siemens Healthcare AG, Lausanne, Switzerland; 7 Division of Dental Biomaterials, Clinic of Reconstructive Dentistry, Center of Dental Medicine, University of Zurich, Zurich, Switzerland; 8 Departement of Reconstructive Dentistry and Gerodontology, School of Dental Medicine, University of Bern, Bern, Switzerland

**Keywords:** Anatomy, Medical research

## Abstract

This prospective study aimed to present, compare, and evaluate the suitability of five different magnetic resonance imaging (MRI) protocols (3D double-echo steady-state (DESS), 3D fast spin echo short-tau inversion recovery (SPACE-STIR), 3D fast spin echo spectral attenuated inversion recovery (SPACE-SPAIR), volumetric interpolated breath-hold examination (T1-VIBE-Dixon), and ultrashort echo time (UTE)) and for orthopantomogram (OPG)-like MRI reconstructions using a novel mandibular coil. Three readers assessed MR-OPGs of 21 volunteers regarding technical image quality (4, excellent; 0, severely reduced), susceptibility to artifacts (3, absence; 0, massive), and visualization of anatomical structures in the oral cavity and surrounding skeletal structures (4, fine details visible; 0, no structures visible). Average image quality was good (3.29 ± 0.83) for all MRI protocols, with UTE providing the best image quality (3.52 ± 0.62) and no to minor artifacts (2.56 ± 0.6). Full diagnostic interpretability of the osseous structures is best in VIBE-Dixon and UTE MR-OPGs. DESS provided excellent visualization of the finest details of the nervous tissue (3.95 ± 0.22). Intra-reader and inter-reader agreement between the readers was good to excellent for all protocols (ICCs 0.812–0.957). MR-OPGs provide indication-specific accurate imaging of the oral cavity and could contribute to the early detection of pathologies, staging, and radiological follow-up of oral and maxillofacial diseases.

## Introduction

An orthopantomogram (OPG) provides a two-dimensional overview image of the dento-maxillofacial complex, including the teeth and adjacent skeletal structures. It enables the radiological assessment of various anatomical structures and pathological conditions in operative dentistry and oral and maxillofacial surgery. OPG, which captures the dento-maxillofacial complex in a single image with a relatively short exposure time and radiation dose (4–30 μSv)^1^, is increasingly being performed to complement the clinical examination for the initial assessment of the patient’s overall dental condition and facial skeleton, diagnosis of possible dental and temporomandibular joint disease or traumatic injury, and planning of third molar surgery, implant therapy, and orthodontic procedures^2–4^. Given the excellent hard tissue contrast of conventional radiographic OPGs and the wide range of diagnostic indications, OPGs have become an integral part of dental education and are considered the most familiar imaging technique in daily dental practice^5^.

Although it is the most commonly performed X-ray-based examination in industrialized countries, this imaging modality has the limitation of inadequately depicting soft tissues and more complex disorders^5^. This is especially relevant for surgical procedures in the vicinity of the inferior alveolar and lingual nerve, whose postoperative temporary disturbances are usually the result of iatrogenic damage from dentoalveolar surgery^6^. Furthermore, radiation exposure from dental radiographs is associated with a 1.46-fold relative increase in the lifetime risk of radiation-induced cancers in radiosensitive, genetically susceptible adolescents^7^, leading in particular to an increased risk of thyroid cancer and meningiomas^8,9^.

In contrast to conventional X-ray-based imaging modalities, ionizing radiation-free magnetic resonance imaging (MRI) with its superior soft tissue contrast, provides an excellent quantitative and qualitative assessment of nervous tissue^10^ but encounters several challenges in depicting bone structures. Cortical bone and dental hard tissue, with its small molecular fraction of hydrogen nuclei and its rapid signal decay after excitation, provides suboptimal signals with conventional MRI protocols such as fast spin echo (FSE) or gradient echo (GE)^11^. However, MRI sequences, such as fast spin echo short-tau inversion recovery (SPACE-STIR)^12^ and double-echo steady-state (DESS)^13,14^, or ultrashort echo time (UTE) sequences^15^ revealed new diagnostic possibilities for the dental field by providing high-contrast resolution imaging, where the generated MRI signals can be digitized and combined for simultaneous imaging of differentially mineralized hard and soft tissues.

Because OPGs in adolescents are primarily needed to visualize the positional relationship between the third molar and the inferior alveolar nerve canal, MR-OPGs emphasize the need for visualization of nerve tissue for preoperative imaging in high-risk surgical procedures, as they could potentially provide by the application of specific imaging protocols beneficial information for the surgeon^16^. Results in the literature show that DESS and SPACE-STIR MRI sequences provide excellent visualization of the thinnest extracranial peripheral nerve branches with a high signal-to-noise ratio (SNR)^16^. On DESS sequences, the branches of the mandibular division of the trigeminal nerve are presented as structures with high signal intensity. DESS combines the signals from FISP and the PSIF echoes, which increases T2 specificity, reduces signal decay due to dephasing, and contrast adjacent anatomic tissue due to the surrounding myelin sheath^17^. On the other hand, UTE type sequences allow for visualization of cortical bone and adjacent tissue through ultrashort hard pulse excitation and three-dimensional center-out radial sampling, providing image quality equivalent to standard pulse sequences^15^. Due to its ultrashort echo time, the UTE sequence is particularly suited to image bone and teeth and reduce metal or field inhomogeneity artifacts.

In clinical routine, MRI of the oral cavity is challenged by motion artifacts, complex and small vascular and neuronal anatomy, and susceptibility induced artifacts due to magnetic field inhomogeneities caused by e.g. dental implants or dentures^18^. Given the recent advances in sequences and innovations such as intraoral coils^19^ or mandibular coils that might allow faster imaging^20^, dental MRI represents a promising option with great potential to be established as a diagnostic tool in various disciplines of operative dentistry and oral and maxillofacial surgery.

Therefore, this prospective feasibility study is relevant for OPG imaging using a novel mandibular coil and comparing and evaluating selected MRI protocols (DESS, SPACE-STIR, SPACE-SPAIR, VIBE, and UTE) from the literature with promising results in dentomaxillofacial imaging in terms of image quality, artifact assessment, and diagnostic accuracy in the specific anatomy of the oral cavity and surrounding skeletal structures. The aim is to determine the advantages and limitations of each sequence to make indication-specific recommendations for clinical practice in oral and maxillofacial radiology.

## Materials and methods

### Study design

This cohort study was conducted in collaboration between the Department of Neuroradiology of the University of Zurich and the Clinic of Cranio-Maxillofacial and Oral Surgery of the University of Zurich and included 21 volunteers. Recruitment of the research participants was performed from August 2022 to November 2022. Enrolled participants underwent MRI examinations, with the data acquisition being performed by trained clinical staff. The gender ratio was 15 males (71%) to 6 females (29%), and the mean age of the cohort group was 34.86 ± 12 years (median age, 31 years; age range, 22–67 years).

The following criteria were required for the study participants to be included: (1) male and female patients aged 18 to 70 years; (2) clinically asymptomatic for head and neck pathologies. The exclusion criteria were: (3) history of recent cranio-maxillofacial and oral surgical procedures (in the last three months), (4) acute odontogenic infection; (5) nerve damage to the three branches of the trigeminal nerve; (6) pregnancy; (7) claustrophobia; (8) standard contraindications to MR imaging, such as metallic intraocular foreign bodies, or cardiovascular implantable electronic devices.

The trial (2022-D0090) was approved by the Cantonal Ethics Commission of Zurich (Switzerland)*.* All volunteers were informed and provided written informed consent to take part in the study according to the Declaration of Helsinki and its later revised ethical standards.

### MRI data acquisition

**Figure 1:**
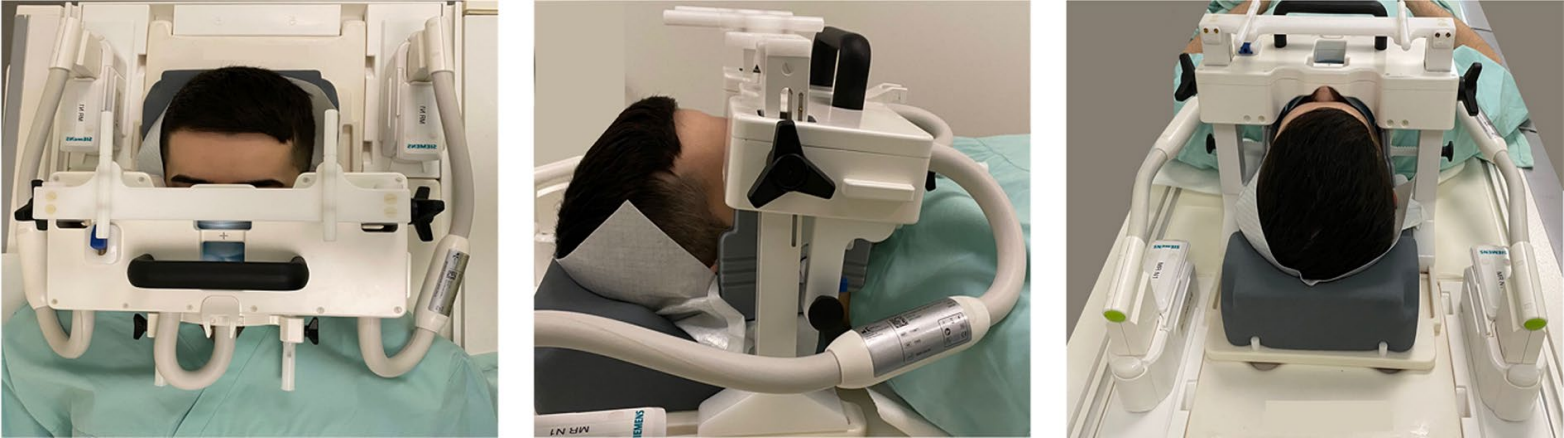
All study participants underwent 3 Tesla MRI (Skyra, release VE11e, Siemens Healthineers, Erlangen, Germany) using a dedicated 15-channels mandibular coil (NORAS MRI products, Hoechberg, Germany). The mandibular coil used in this study has a field of view of 32 × 16 × 16 cm^3^ and is an optimized 14+ 1 receiver coil array and positioning system specifically designed for high-resolution imaging of dental structures in the oral cavity.

All study participants underwent MRI at 3-Tesla (MAGNETOM Skyra, release VE11E, Siemens Healthineers, Erlangen, Germany) with gradient specifications 45 mT/m and 200 T/m/s using a dedicated 15-channel mandibular coil (NORAS MRI products, Hoechberg, Germany). The mandibular coil used in this study has a field of view of 32 × 16 × 16 cm and is an optimized 14+ 1 receiver coil array and positioning system specifically designed for high-resolution imaging of maxillomandibular structures. It consists of a curved 12 × 38 cm^2^ phased array coil with 14 elements between two bars. Fixation elements allow precise positioning of the patient’s head, while openings are provided for the nose and mouth. The central junction between the two openings should be located directly above the upper lip. The outer wings of the array coil are designed to be flexible and can be freely and precisely adapted to the patient's own mandibular anatomy. In addition, a mirror and head fixation can be used to increase patient comfort, reduce motion artifacts, and minimize distress for claustrophobic patients^21^ (Fig. 1).

The acquired sequences were 3D-DESS, 3D-SPACE-STIR, and 3D-SPACE-SPAIR for soft tissue contrast and 3D T1-VIBE-Dixon and a 3D UTE prototype sequence for bone and teeth imaging. Two fat saturation methods (STIR and SPAIR) for the SPACE sequence were compared, but all other parameters were kept equal. All sequences were acquired with sub-millimeter isotropic resolution and optimized for quality a pilot. Detailed sequences’ parameters are reported in Table 1.

**Table 1:** The acquired sequences were 3D double-echo steady-state (DESS), 3D fast spin echo short-tau inversion recovery (SPACE-STIR), 3D fast spin echo spectral attenuated inversion recovery (SPACE-SPAIR), 3D volumetric interpolated breath-hold examination (T1-VIBE-Dixon), and 3D ultrashort echo time (UTE).

	DESS	T2 SPACE STIR	T2 SPACE SPAIR	T1 Vibe Dixon	UTE
Sequence name	de_rr	spcir	spc	fl	WIP_fl
Geometry					
Dimension of acquisition	3D	3D	3D	3D	3D radial
Orientation	semi-axial	semi-axial	semi-axial	coronal	semi-axial
Phase enc. dir.	R >> L	A >> P	A >> P	F >> H	A >> P
FoV read and phase [mm]	242 × 242	190 × 190	190 × 190	380 × 212	230 × 230
Slices per slab	104	120	120	96	384
Acq. slice thickness [mm]	0.75	0.75	0.75	1.00	0.6
Phase/slice oversampling	0%/100%	35%/20%	35%/20%	40%/67%	–
Acq. matrix read/phase	320 × 320	256 × 256	256 × 256	380 × 380	384 × 384
Radial views	–	–	–	–	40’000
Voxel recon size [mm^3^]	0.38×0.38×0.75	0.37×0.37×0.75	0.37×0.37×0.75	0.8×0.8×1.0	0.6×0.6×0.6
Contrast					
Flip angle [degree]	30	T2 var	T2 var	11	5
Echo spacing [ms]	–	4.82	4.82	–	–
Echo train duration [ms]	–	284	284	–	–
TR [ms]	11.16	3300	3300	5.81	4.62
TE [ms]	4.21	113	115	2.46/3.69	0.04
Magn. preparation	off	Non-sel. IR	None	–	–
TI [ms]	–	220	–	–	–
Fat suppr.	Water excit. normal	None	SPAIR, strong	Dixon,optim inphase	None
Duration					
TA [min:s]	12:24	12:36	12:36	05:28	03:07
Averages	1	1.4	1.4	1	1
PAT total accel. factor	Off	4	4	Off	0
PAT mode	–	GRAPPA phase 2, ref.l. 24 slice 2, ref.l. 24	GRAPPA phase 2, ref.l. 24 slice 2, ref.l. 24	–	–
Asymmetric echo	Off	–	–	weak	strong
Bandwidth [Hz/Px]	355	425	425	660/700	1184
Artifact reduction					
Flow comp.	Read	No	No	–	–
Excitation	Slab-sel.	Slab-sel.	Slab-sel.	Slab-sel.	Non-sel.
RF spoiling	–	–	–	On	On
Incr. gradient spoiling	–	–	–	On	On
Distortion corr.	2D	3D	3D	3D	–

Two fat saturation methods (STIR and SPAIR) for the SPACE sequence were compared, but all other parameters were kept equal. All sequences were acquired with sub-millimetric isotropic resolution and optimized for quality in a pilot study. Detailed sequence parameters are provided.

### Image analysis

MRI data was stored and evaluated in the local Picture Archiving and Communication System (PACS) (IMPAX EE R20, release XV, Agfa Healthcare, Mortsel, Belgium) using a 2-megapixel high quality liquid-crystal display. To create orthopantomogram-like MR images with a slice thickness of 0.5 mm, image post-processing was performed in syngo.via (release VB60A, Siemens Healthineers, Erlangen, Germany) using the curved multiplanar reconstruction (MPR) function. All MR-OPGs of the five different MR protocols were created with the same slice thickness for better comparability and standardization, and sequential curvilinear reconstructions were evaluated. MR-OPG reconstructions were created manually, by a single calibrated examiner (A.A.H.), by performing the reconstruction planning through the occlusal plane.

MR-OPG’s from the DESS, SPACE-STIR, SPACE-SPAIR, T1-VIBE-Dixon, and UTE datasets (21 participants, each of whom underwent all 5 MRI protocols, resulting in 105 datasets) were evaluated in randomized order by three readers with varying degrees of experience (reader A, attending board-certified oral surgeon; reader B, resident oral surgeon; reader C, attending board-certified radiologist and neuroradiologist). Prior to the assessment, a calibration session was conducted by all three readers, in which four random cases were evaluated together to resolve any uncertainties. Inter-reader and intra-reader reliability was assessed. The readers were blinded to each other’s results and to their previous readouts. Intra-reader agreement was examined by having Reader B, the least experienced reader with 3 years of experience, and Reader C, the most experienced reader with 6 years of experience in general radiology and 30 years of experience in neuroradiology, repeat the evaluation after a time interval of at least 3 weeks to avoid recall bias. The intra-reader agreement of reader B was selected to determine the expected lower limit for non-experts in MRI reading to assess its applicability in routine daily dental practice. Qualitative analysis was performed using a modified Likert rating scale to evaluate overall technical image quality, assess artifacts, and evaluate visualization of specific anatomical structures in the oral cavity and surrounding skeletal structures relevant to OPG imaging^22^. Bony structures including the osseous delineation of the maxillary sinus, the temporomandibular joint (with emphasis on temporal bone of the cranium and the mandibular condyle), the mandibular angle, and dental structures such as the dental complex (tooth and periapical region), the dental pulp, and the inferior alveolar nerve were assessed.

### Readout

Overall technical image quality was assessed using a modified 5-point Likert scale^23^: 4, excellent image quality with full diagnostic interpretability; 3, good image quality with full diagnostic interpretability; 2, satisfactory image quality and diagnostic interpretability; 1, markedly reduced image quality and impaired diagnostic interpretability; 0, severely reduced image quality, allowing no diagnostic interpretability. Regarding artifact assessment, a 4-point Likert scale was used to rate the presence of motion artifacts, pulsation, and ghosting: 3, absence of artifacts (none); 2, minor artifacts (low); 1, moderate artifacts (moderate); 0, massive artifacts (high). Visualization of the above-mentioned anatomical structures (maxillary sinus, temporomandibular joint, mandibular angle, tooth and periapical region, dental pulp of the second premolar and first molar in each quadrant, and inferior alveolar nerve) was assessed considering a modified Likert Scale according to Sabarudin et al.^22^: 4, fine details are visualized with full diagnostic interpretability; 3, small details are visualized with good diagnostic interpretability; 2, only broad detail visible with impaired diagnostic interpretability; 1, significant structures are not visible, allowing no diagnostic interpretability; 0, no structures are visible, allowing no diagnostic interpretability.

### Statistical analysis

Statistical analyses were performed using IBM SPSS Statistics software (version 26.0, IBM Corp. Armonk, NY, USA). Descriptive statistics were used to analyze the image quality, artifacts assessment and diagnostic accuracy of specific anatomical structures, determining metric variables with means, standard deviations, medians, minima and maxima, and categorical variables with frequencies and percentages. Inter- and intra-reader agreement was analyzed by Intraclass Correlation Coefficient (ICC) type 2:1 and the 95% Confidence Interval (CI) based on absolute agreement 2-way random model. Based on the selected 95%-CI, the ICC values, and thus the agreement beyond chance, can be interpreted as follows: poor, < 0.5; moderate, 0.5–0.75; good, 0.75–0.9; and excellent, > 0.9^24^.

## Results

Intra-reader and inter-reader agreement between the three readers are shown in Table 2. Generally, both intra-reader and inter-reader agreement were generally good to excellent for all MRI sequences, with ICC values up to 0.96. Thereby, the highest values of reliability ICC (95% CI) were observed for DESS (Intra_ICC (95% CI)_ = 0.94 (0.79–0.96), Inter_ICC (95% CI)_ = 0.94 (0.85–0.96); both *p* < 0.001), DIXON (Intra_ICC (95% CI)_ = 0.92 (0.75–0.96), Inter_ICC (95% CI)_ = 0.95 (0.82–0.96); both *p* < 0.001) and UTE (Intra_ICC (95% CI)_ = 0.88 (0.6–0.92), Inter_ICC (95% CI)_ = 0.94 (0.78–0.96); both *p* < 0.001) MR-OPGs. Regarding the average inter-reader agreement, values of MR-OPGs using DESS (ICC = 0.93) and VIBE-Dixon (ICC = 0.93) protocols were excellent, while SPACE-STIR (ICC = 0.81) provided the lowest value, which, however, is still considered good^24^.

**Table 2:** Qualitative assessment of OPG (orthopantomogram) MRI reconstructions (MR-OPG) from the 3D double-echo steady-state (DESS), 3D fast spin echo short-tau inversion recovery (SPACE-STIR), 3D fast spin echo spectral attenuated inversion recovery (SPACE-SPAIR), 3D volumetric interpolated breath-hold examination (T1-VIBE-Dixon), and 3D ultrashort echo time (UTE) datasets were evaluated in randomized order by three readers with varying degrees of experience (reader A, attending board-certified oral surgeon; reader B, resident oral surgeon; reader C, attending board-certified radiologist and neuroradiologist).

Reader agreement	MRI sequence	ICC (95% CI)	p-value
Reader A and B	DESS	0.92 (0.767–0.95)	< 0.001
	SPACE-STIR	0.756 (0.517–0.876)	< 0.001
	SPACE-SPAIR	0.843 (0.651–0.935)	< 0.001
	VIBE-Dixon	0.83 (0.717–0.961)	< 0.001
	UTE	0.865 (0.622–0.938)	< 0.001
Reader B and C	DESS	0.91 (0.654–0.959)	< 0.001
	SPACE-STIR	0.9 (0.659–0.931)	< 0.001
	SPACE-SPAIR	0.863 (0.691–0.943)	< 0.001
	VIBE-Dixon	0.89 (0.677–0.932)	< 0.001
	UTE	0.85 (0.579–0.913)	< 0.001
Reader C and A	DESS	0.957 (0.881–0.979)	< 0.001
	SPACE-STIR	0.856 (0.739–0.921)	< 0.001
	SPACE-SPAIR	0.898 (0.771–0.959)	< 0.001
	VIBE-Dixon	0.931 (0.817–0.968)	< 0.001
	UTE	0.902 (0.672–0.932)	< 0.001
Reader B1 and B2	DESS	0.937 (0.793–0.961)	< 0.001
	SPACE-STIR	0.715 (0.594–0.916)	< 0.001
	SPACE-SPAIR	0.764 (0.687–0.942)	< 0.001
	VIBE-Dixon	0.921 (0.751–0.96)	< 0.001
	UTE	0.882 (0.599–0.919)	< 0.001
Reader C1 and C2	DESS	0.944 (0.847–0.957)	< 0.001
	SPACE-STIR	0.833 (0.795–0.962)	< 0.001
	SPACE-SPAIR	0.86 (0.781–0.948)	< 0.001
	VIBE-Dixon	0.953 (0.821–0.96)	< 0.001
	UTE	0.94 (0.78–0.96)	< 0.001
Average and standard deviation	DESS	0.934	0.017
	SPACE-STIR	0.812	0.067
	SPACE-SPAIR	0.846	0.045
	VIBE-Dixon	0.905	0.043
	UTE	0.888	0.031

Intra- and inter-reader agreement was evaluated by Intraclass Correlation Coefficient (ICC): type 2:1 and the 95% Confidence Interval (CI) based on absolute agreement 2-way random model. Corresponding p-values are provided.

**Figure 2:**
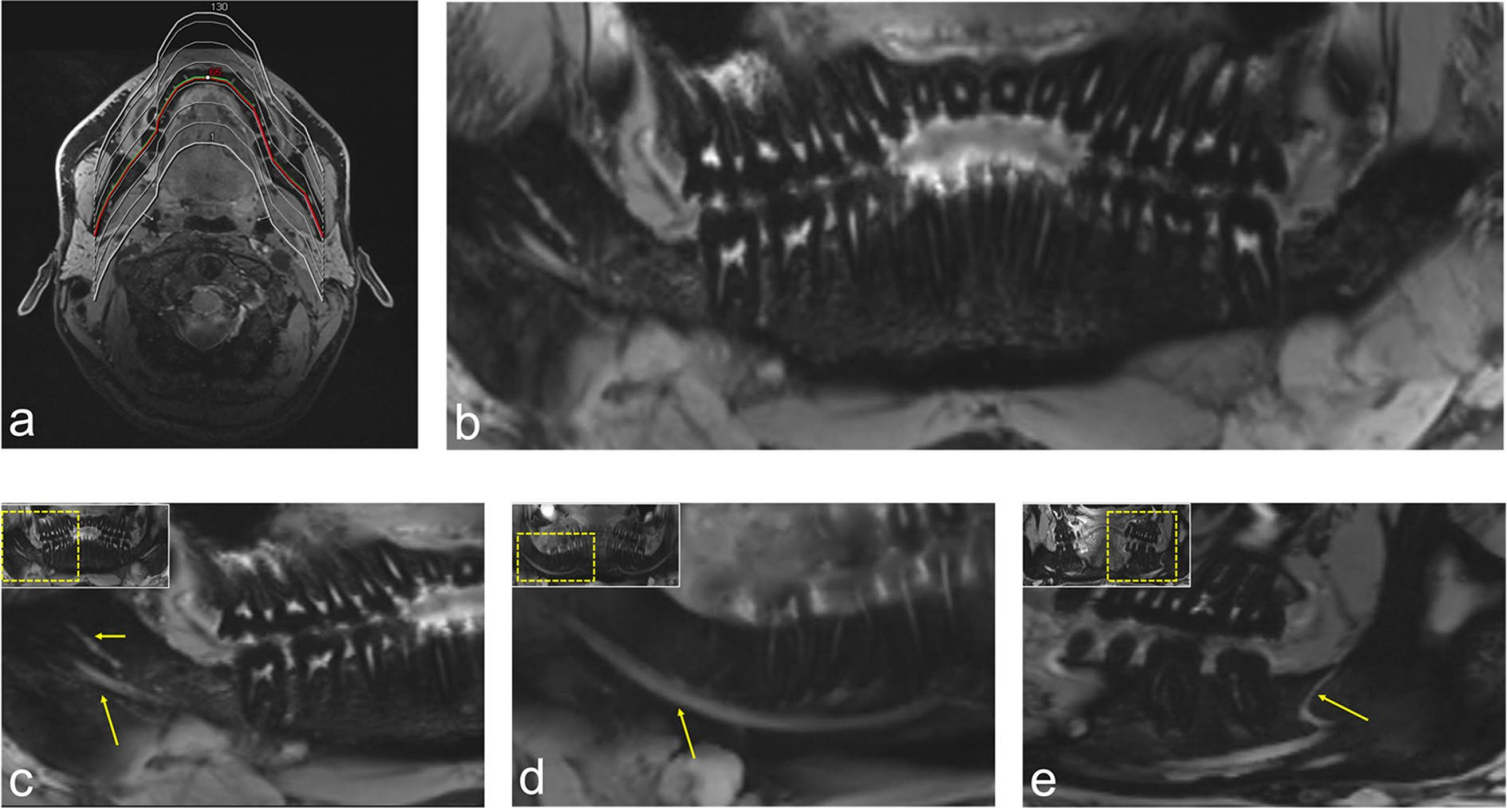
OPG (orthopantomogram) MRI reconstructions (MR-OPG) from a 3D double-echo steady-state (DESS) dataset. T2-w imaging using DESS-MRI (scan time approximately 12 minutes) allows visualization of the complex neural microarchitecture of the thinnest peripheral branches of the mandibular division of the trigeminal nerve. (**a**) MR-OPG with a slice thickness of 0.5 mm were created using the curved multiplanar reconstruction (MPR) function of syngo.via (release VB60a, Siemens Healthineers, Erlangen, Germany). (**b**) Overview image of a DESS MR-OPG. (**c**) Visualization of the fourth quadrant of a study participant’s DESS MR-OPG. The long arrow points to the inferior alveolar nerve (IAN), while the short arrow represents the lingual nerve. (**d**) Visualization of the course of the T2- weighted hyperintense signal of the IAN through the mandible, whereas (**e**) shows a retromolar branch of the IAN.

**Figure 3:**
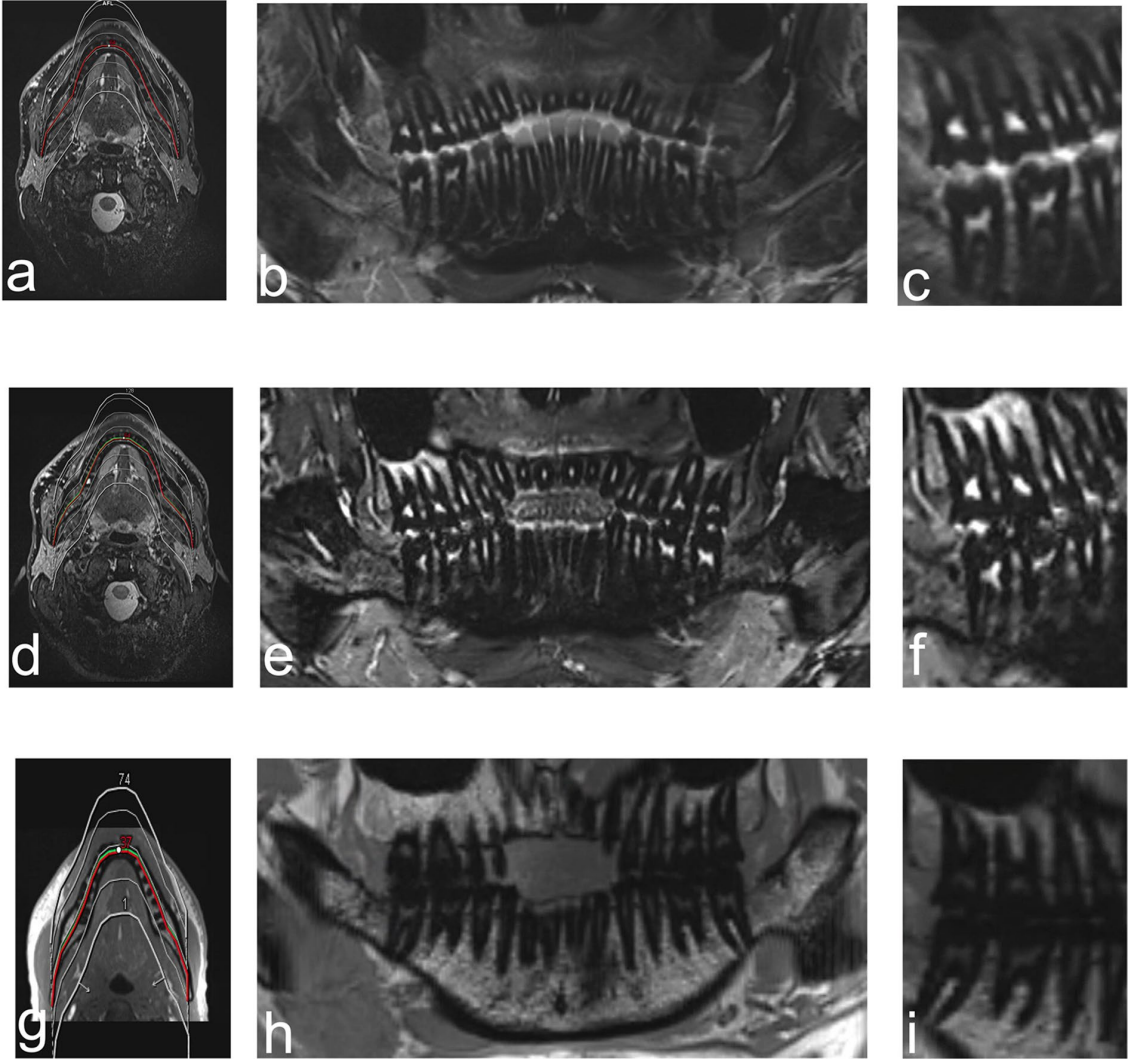
OPG (orthopantomogram) MRI reconstructions (MR-OPG) from (**a**–**c**) 3D fast spin echo short-tau inversion recovery (SPACE STIR), (**d**–**f**) 3D fast spin echo spectral attenuated inversion recovery (SPACE-SPAIR), and (**g**–**i**) volumetric interpolated breath-hold examination (T1-VIBE-Dixon) datasets. (**a**), (**d**) and (**g**) show the planning of the MR-OPG images. Orthopantomogram-like MR images using (**b**) (SPACE-STIR), (**e**) (SPACE-SPAIR), and (**h**) (VIBE-Dixon) sequences. (**c**), (**f**) and (**i**) visualize the anatomy of teeth in (**c**) (SPACE-STIR), (**f**) (SPACE-SPAIR), and (**i**) (VIBE-Dixon) MRI.

**Figure 4:**
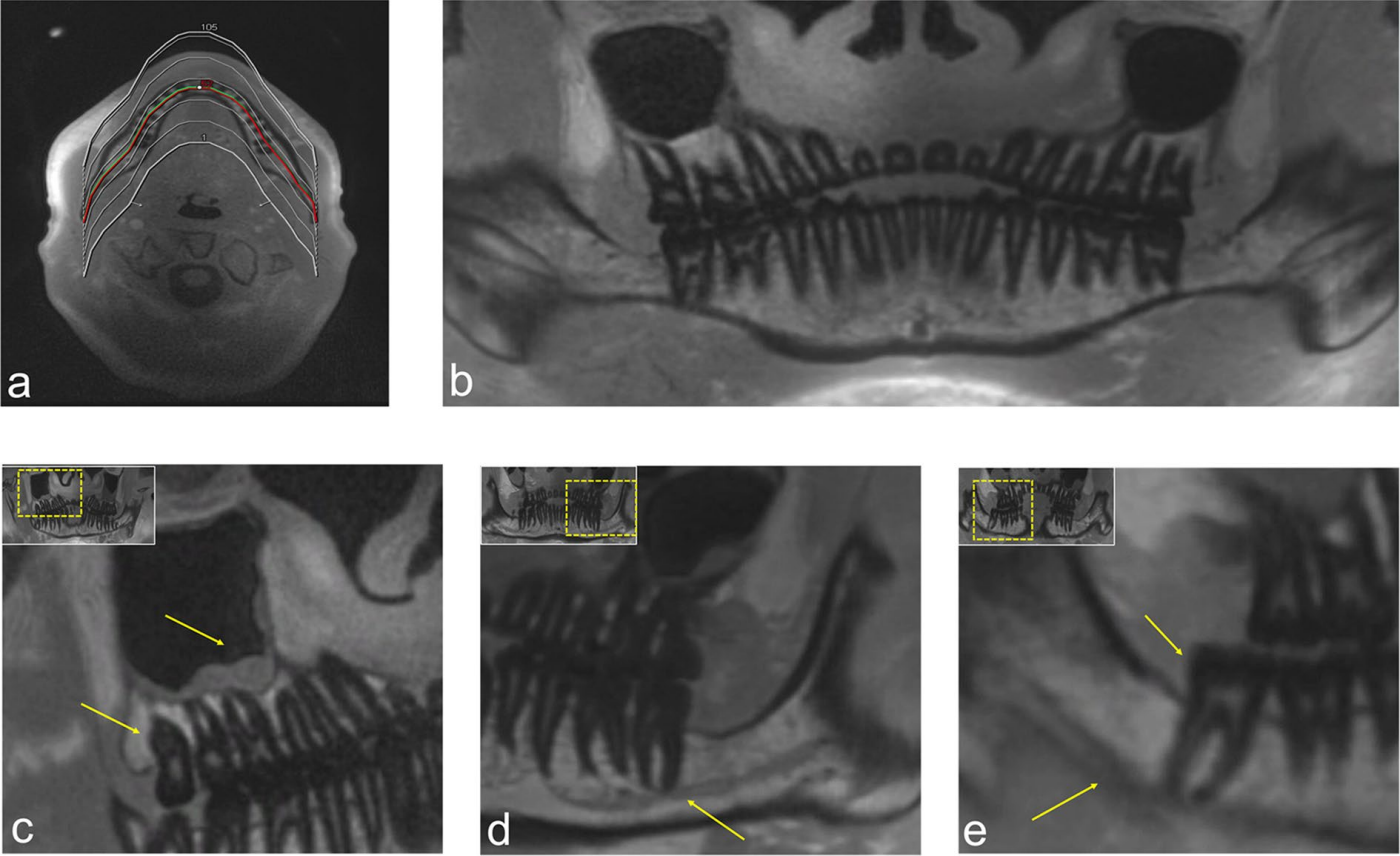
OPG (orthopantomogram) **-** like MRI reconstructions (MR-OPGs) from a 3D ultrashort echo time (UTE) prototype sequence for bone and teeth imaging. (**a**) The planning of the orthopantomogram-like MR images. (**b**) Overview image of a UTE MR-OPG. (**c**) Visualization of the positional relationship of the maxillary third molar in the first quadrant and the maxillary sinus, with a retention cyst. (**d**) Visualization of the course of the hypointense inferior alveolar nerve (IAN) within the inferior alveolar canal through the mandible. (**e**) Shows the positional relationship between the mandibular third molar and the IAN in UTE MR-OPG.

The average image quality score was overall good for the various MRI protocols (3.29 ± 0.83), with UTE providing the best image quality (3.52 ± 0.62). Regarding artifacts caused by the presence of motion artifacts, pulsation, and ghosting, generally no to minor artifacts were present, while the lowest artifacts score was observed in UTE (2.56 ± 0.6). Visualization of the bony boundaries of the maxillary sinus is best represented in VIBE-Dixon (3.9 ± 0.44) and UTE (3.86 ± 0.48) MR-OPGs with full diagnostic interpretability, whereas all black bone MRI sequences (DESS, SPACE-STIR, or SPACE-SPAIR) allowed inferior visualization in comparison. Comparable results were also found for visualization of the mandibular angle and the temporomandibular joint, with higher values for the mandibular angle on black bone MRI, whereas good visualization of the temporomandibular joint was mainly observed in DESS. Regarding the nervous tissue, DESS MRI provided excellent visualization of finest details of the inferior alveolar nerve and its branches (3.95 ± 0.22) (Fig. 2), while SPACE-SPAIR MRI provided the best visualization of the dental pulp (3.8 ± 0.7) (Fig. 3). In contrast, the MR-OPGs of VIBE-Dixon (3.57± 0.81) and UTE (3.62 ± 0.67) achieved the best results in imaging the teeth and periapical regions (Fig. 4). For more information see Table 3.

**Table 3:** Qualitative assessment of OPG (orthopantomogram) MRI reconstructions (MR-OPG’s) from the 3D double-echo steady-state (DESS), 3D fast spin echo short-tau inversion recovery (SPACE-STIR), 3D fast spin echo spectral attenuated inversion recovery (SPACE-SPAIR), 3D volumetric interpolated breath-hold examination (T1-VIBE-Dixon), and 3D ultrashort echo time (UTE) datasets were evaluated in randomized order by three readers with varying degrees of experience (reader A, attending board-certified oral surgeon; reader B, resident oral surgeon; reader C, attending board-certified radiologist and neuroradiologist).

	MRI sequence	Reader A	Reader B1	Reader B2	Reader C1	Reader C2	Average
Image quality	DESS	3.29 ± 1.15	3.19 ± 1.17	3.17 ± 1.1	3.3 ± 1.05	3.29 ± 1.15	3.25 ± 1.12
	SPACE-STIR	3.24 ± 0.94	3.14 ± 0.96	3.24 ± 0.94	3.12 ± 0.94	3.09 ± 0.94	3.17 ± 0.94
	SPACE-SPAIR	3.3 ± 0.87	3.26 ± 0.87	3.3 ± 0.47	3.17 ± 0.65	3.2 ± 0.72	3.25 ± 0.72
	VIBE-Dixon	3.24 ± 0.83	3.29 ± 0.72	3.31 ± 0.78	3.29 ± 0.78	3.23 ± 0.59	3.27 ± 0.74
	UTE	3.52 ± 0.6	3.5 ± 0.63	3.61 ± 0.86	3.5 ± 0.6	3.48 ± 0.42	3.52 ± 0.62
Artifacts	DESS	2.14 ±0.9	2.19 ± 0.82	2.29 ± 0.85	2.29 ± 0.85	2.2 ± 0.58	2.22 ± 0.8
	SPACE-STIR	2.24 ± 0.7	2.19 ± 0.75	2.08 ± 0.69	2.29 ± 0.72	2.33 ± 0.73	2.23 ± 0.72
	SPACE-SPAIR	2.4 ± 0.68	2.37 ± 0.68	2.21 ± 0.39	2.4 ± 0.68	2.51 ± 0.45	2.38 ± 0.58
	VIBE-Dixon	2.38 ± 0.67	2.48 ± 0.59	2.38 ± 0.39	2.33 ± 0.66	2.4 ± 0.29	2.39 ± 0.52
	UTE	2.62 ± 0.59	2.55 ± 0.61	2.57 ± 0.59	2.52 ± 0.6	2.55 ± 0.61	2.56 ± 0.6
Maxillary sinus	DESS	3.62 ± 0.74	3.52 ± 0.75	3.57 ± 0.75	3.47 ± 0.66	3.57 ± 0.75	3.55 ± 0.73
	SPACE-STIR	3.57 ± 0.68	3.48 ± 0.68	3.52 ±0.68	3.52 ± 0.68	3.52 ± 0.68	2.52 ± 0.68
	SPACE-SPAIR	3.6 ± 0.68	3.58 ± 0.69	3.6 ± 0.68	3.6 ± 0.68	3.6 ± 0.68	3.6 ± 0.68
	VIBE-Dixon	3.9 ± 0.44	3.9 ± 0.44	3.9 ± 0.44	3.9 ± 0.44	3.9 ± 0.44	3.9 ± 0.44
	UTE	3.9 ± 0.44	3.85 ± 0.49	3.86 ± 0.48	3.81 ± 0.51	3.86 ± 0.48	3.86 ± 0.48
Temporomandibular joint	DESS	3.8 ± 0.52	3.79 ±0.54	3.8 ± 0.52	3.8 ± 0.52	3.8 ± 0.52	3.8 ± 0.52
	SPACE-STIR	2.84 ± 0.69	2.89 ±0.83	2.89 ±0.81	2.95 ± 0.78	2.89 ± 0.81	2.89 ± 0.78
	SPACE-SPAIR	2.84 ± 0.5	2.94 ± 0.64	2.95 ± 0.62	2.95 ± 0.62	2.89 ± 0.58	2.91 ± 0.59
	VIBE-Dixon	3.42 ± 1.24	3.73 ± 0.65	3.69 ± 0.67	3.73 ± 0.65	3.73 ± 0.65	3.66 ± 0.77
	UTE	3.95 ± 0.22	3.89 ± 0.32	3.9 ± 0.3	3.9 ± 0.31	3.89 ± 0.32	3.91 ± 0.29
Mandibular angle	DESS	3.62 ± 0.81	3.62 ± 0.81	3.62 ± 0.81	3.62 ± 0.81	3.62 ± 0.81	3.62 ± 0.81
	SPACE-STIR	2.84 ± 0.69	2.89 ± 0.83	2.89 ± 0.81	2.95 ± 0.78	2.91 ± 0.74	2.9 ± 0.77
	SPACE-SPAIR	3.4 ± 0.82	3.37 ± 0.83	3.4 ± 0.8	3.4 ± 0.82	3.4 ± 0.82	3.39 ± 0.82
	VIBE-Dixon	3.71 ± 0.71	3.76 ± 0.7	3.76 ± 0.7	3.76 ± 0.7	3.76 ± 0.7	3.75 ± 0.7
	UTE	3.9 ± 0.44	3.9 ± 0.44	3.9 ± 0.44	3.9 ± 0.44	3.9 ± 0.44	3.9 ± 0.44
Tooth and periapical region	DESS	2.86 ± 1.06	2.81 ± 1.12	2.9 ± 1.09	2.86 ± 1.1	2.9 ± 1.09	2.87 ± 1.09
	SPACE-STIR	2.76 ± 0.83	2.62 ± 0.81	2.71 ± 0.85	2.71 ± 0.85	2.68 ± 0.84	2.7 ± 0.84
	SPACE-SPAIR	3.15 ± 0.81	3.16 ± 0.83	3.15 ± 0.81	3.1 ± 0.85	3.18 ± 0.79	3.15 ± 0.82
	VIBE-Dixon	3.57 ± 0.81	3.57 ±0.81	3.57± 0.81	3.57± 0.81	3.57± 0.81	3.57± 0.81
	UTE	3.67 ± 0.66	3.6 ± 0.68	3.62± 0.67	3.62 ± 0.67	3.62 ± 0.67	3.62 ± 0.67
Dental pulp	DESS	3.24 ± 0.94	3.14 ± 0.96	3.19 ± 0.98	3.21 ± 0.49	3.24 ± 0.94	3.2 ± 0.86
	SPACE-STIR	3.62 ± 0.81	3.48 ± 0.87	3.62 ± 0.81	3.57 ± 0.74	3.62 ± 0.81	3.58 ± 0.81
	SPACE-SPAIR	3.8 ± 0.7	3.79 ± 0.71	3.8 ± 0.7	3.8 ± 0.7	3.8 ± 0.7	3.8 ± 0.7
	VIBE-Dixon	2.29 ± 0.64	2.43 ± 0.75	2.29 ± 0.64	2.29 ± 0.56	2.31 ± 0.69	2.32 ± 0.66
	UTE	2.76 ± 0.64	2.75±0.716	2.81 ± 0.75	2.71 ± 0.78	2.86 ± 0.73	2.78 ± 0.72
Inferior alveolar nerve	DESS	3.95 ± 0.22	3.95 ± 0.22	3.95 ± 0.22	3.95 ± 0.22	3.95 ± 0.22	3.95 ± 0.22
	SPACE-STIR	3.76 ± 0.54	3.76 ± 0.54	3.76 ± 0.54	3.76 ± 0.54	3.76 ± 0.54	3.76 ± 0.54
	SPACE-SPAIR	3.85 ± 0.49	3.79 ± 0.63	3.8 ± 0.62	3.85 ± 0.49	3.85 ± 0.49	3.83 ± 0.54
	VIBE-Dixon	3.33 ± 0.66	3.33 ± 0.67	3.33 ± 0.66	3.33 ± 0.66	3.33 ± 0.66	3.33 ± 0.66
	UTE	3.52 ± 0.68	3.45 ± 0.69	3.52 ± 0.68	3.52 ± 0.68	3.48 ± 0.68	3.5 ± 0.68

A modified Likert rating scale was used to evaluate overall technical image quality (4, excellent; 0, severely reduced image quality), assess artifacts (3, absence of artifacts; 0, massive artifacts), and evaluate visualization of specific anatomical structures in the oral cavity and surrounding skeletal structures relevant to OPG imaging (4, fine details are visible; 0; no structures are visible).

## Discussion

The findings of this study support the hypothesis that recent advances in MRI technology using high-field MR scanners, specifically implemented and optimized sequences, and newly developed hardware such as mandibular coils, enable the acquisition of OPG-like MR images that enable simultaneous visualization of hard and soft biological tissues in routine clinical settings with a far better resolution than x-ray based OPG. By using black bone MRI sequences such as DESS, SPACE-STIR, or SPACE-SPAIR, the “black bone” can be clearly delineated from the adjacent soft tissues, resulting in improved soft tissue/bone contrast and thus providing, among other benefits, excellent visualization of nervous tissue. In contrast, high-field MRI combining UTE imaging with radial signal sampling techniques provided robust images of hard tissue, including cortical bone and teeth, with superior diagnostic accuracy. These evaluations were very robust and excellently reproducible between observers.

T2-w images are favored for assessing regular nerve anatomy, as well as for diagnosing of the majority of pathological alterations^25^. In general, the ideal image plane for assessing peripheral nerves is perpendicular to their long axis, making an isotropic 3D data acquisition followed by multiplanar image reconstruction the preferred diagnostic approach^26^. DESS-OPG provided high diagnostic confidence in identifying the inferior alveolar nerve as a high-intensity structure reflecting T2/T1 weighting from the PSIF echo signal with high inter- and intra-reader reliability, confirming previously published results^13,27,28^ and allowing visualization of its branches and fascicular bundles in almost all cases. In addition to the benefit of more accurate and individualized preoperative planning of surgical procedures in the vicinity of the inferior alveolar nerve, this diagnostic tool could facilitate the detection of fascicle discontinuity, focal size changes of the inferior alveolar nerve, trace deviations, and the presence of other nerve-related pathologies, such as various subtypes of neuromas. However, DESS MRI cannot differentiate between neural and vascular tissue of the inferior alveolar neurovascular bundle within the mandibular canal, indicating its limitation^29^. The high SNR of DESS MRI can be explained by combination on a pixel-by-pixel basis of the signals generated by the FID-like and Echo-like signals from the steady-state free precession. However, the PSIF/echo contribution makes DESS susceptible to motion artifacts^30^, which was only partially observed in this study, as the mandibular coil allows an excellent fixation of the patient’s head and jaw. Nevertheless, tongue and deglutition movements are inherently difficult to control.

Given the fat suppression technique in the SPACE-STIR and SPACE-SPAIR sequences, acquired primarily for characterization of nervous tissues, they were also able to accurately depict soft tissues, albeit with somewhat lower diagnostic accuracy and inter-rater reliability, while SPACE-SPAIR, in particular, provided excellent visualization of the dental pulp and periodontal tissue. In comparing two fat saturation techniques for the SPACE sequence, STIR and SPAIR, both are used in MRI imaging for fat signal suppression, which can interfere with the visualization of specific structures of interest. STIR nulls signal from both fat and water and recovers water signal, while SPAIR uses a spectrally selective inversion pulse to null only the fat signal. STIR is less susceptible to boundary zone and metal artifacts, while SPAIR is less sensitive to field inhomogeneity^31^. Nonetheless, SPAIR can result in boundary zone artifacts that can negatively impact diagnostic accuracy, particularly in dental MRI, which significantly limits the use of SPACE-SPAIR in specific patient populations. However, the proximity of the blood vessels to the nervous tissue, combined with the use of a fat suppression technique as used in SPACE-STIR MRI, imposes some limitations, as the conspicuous hyperintense vascular signal may potentially interfere with the visualization of the inferior alveolar nerve within the neurovascular bundle, limiting its diagnostic accuracy^10^. Therefore, recent research suggests the suppression of vascular signals by adding a motion-sensitizing T2 preparation prepulse, resulting in a higher contrast-to-noise ratio (CNR) without significantly decreasing SNR in anatomical regions with a close proximity of nerves to vascular structures^32^. On the other hand, Casselman et al. showed that cranial nerve imaging (3D CRANI), a novel high-field STIR TSE sequence, with gadolinium contrast administration significantly improved suppression quality and nerve visualization in the assessment of extraforaminal cranial nerves^33^. Thus, robust blood vessel suppression, better image quality and fewer artifacts could be achieved for magnetic resonance neurography in various anatomical regions. However, SPACE-STIR protocol in the third molar region allowed the distinction between nervous and vascular tissue of the inferior alveolar neurovascular bundle within the mandibular canal, with vessels exhibiting stronger signals than nervous structures, providing potentially clinically relevant information for preoperative planning of high-risk surgery^12^. However using SPACE-STIR brings certain disadvantages; despite providing an improved contrast-to-noise ratio for specific lesions, the overall SNR may be suboptimal, confirming previously published results^12^.

To the authors’ knowledge, the only article evaluating MR-OPGs was by Manoliu and colleagues^26^, who proposed a new technique for MR neurographic orthopantomograms by superimposing UTE of the bone and teeth on functional MR neurography. In their study, it was possible to assess the inferior alveolar nerve in all cases by performing fiber tractography with evaluation of quantitative parameters and physiological diffusion properties using a 64-channel phased array coil^26^. However, the main difficulty in MR neurography is the selective and continuous visualization of the thinnest peripheral extracranial nerves with high contrast and resolution. Given the use of three-dimensional variable-flip-angle turbo spin-echo (SPACE) sequence and background suppression in T2-weighted imaging, there have been recent developments that have optimized the clinical protocols^34^. By using the mandibular coil and optimized sequences, the results obtained in this study allow easier and faster acquisition and processing while achieving comparable or even better image quality of the nerves.

In summary, black bone imaging improves the soft tissue to bone contrast by suppressing fat and free water while maintaining a uniform soft tissue background. This optimizes the ability to clearly visualize bony structures in anatomical regions where they are enveloped within soft tissue, such as the mandibular region^35^. However, in anatomical areas where bone is adjacent to air, such as the paranasal sinuses, difficulties remain as both provide low signals in these MRI protocols, making distinction challenging, which was also observed in this study. A tremendous potential has been seen in imaging of benign pathologies in the head and neck region, which often require repeated scanning in young, radiosensitive patients, resulting in a significant reduction in radiation dose constraint over a lifetime^35^.

Compared to the black bone sequences (DESS, SPACE-STIR, and SPACE-SPAIR), UTE and T1-w VIBE-Dixon provided high diagnostic accuracy in anatomical delineation of the maxillary sinus, the temporal bone of the cranium and the mandibular condyle, the mandibular angle, and dental structures. Zero echo time (ZTE) and UTE are very promising tools because of their ability of being able to detect signals from fast decaying short-T2 components in tissue^36^. The use of ZTE and UTE has been increasingly applied in the segmentation and visualization of cranial bones, with the results obtained providing accurate visualization of cranial structures and volumetric measurements showing close agreement with conventional computed tomography data^37^. To the authors’ knowledge, there are a couple of UTE studies in the dental field that indicate its usefulness in visualization of mineralized dental tissue^38^ and in caries diagnosis, while there are still some limitations regarding dental fillings^39^. Our results confirm these findings, as they provided excellent visualization of cranial structures, dental structures, and even accurate anatomical delineation of the inferior alveolar nerve within the mandibular canal with clinically tolerable scan times of less than three minutes. In addition, the T1-w VIBE-Dixon protocol could provide different information due to the acquired in-phase and out-of-phase images and the generation of water-only and fat-only images.

The presented technique of MR-OPGs, which uses specific MRI sequences and a dedicated mandibular coil, provides an overview image of the dentoalveolar structures and adjacent skeletal areas. This enables radiological assessment of numerous dental issues from a cariological, endodontic, periodontal, and oral surgical perspective. UTE MR-OPGs have the potential to provide a comprehensive assessment of high-resolution anatomical visualization of hard tissues, comparable to conventional radiographic-based modalities, with a scan time of three minutes. UTE MRI shows promise for surgical teeth extraction, apicoectomy, and orthodontic treatment planning. VIBE-DIXON MR-OPGs also have comparable potential for the same procedures, but with a scan time approximately twice as long. They are potentially suitable for clinical use in the detection and differential diagnosis of space-occupying lesions. However, T2-weighted imaging is favored for high-resolution visualization of neural tissue, specifically using the DESS protocol (scan time about 12 min). This protocol enables visualization of the complex neural microarchitecture of the thinnest peripheral branches of the mandibular division of the trigeminal nerve. This could be helpful in various oral and maxillofacial surgical procedures performed near the inferior alveolar or lingual nerve, such as third molar surgery, dental implant planning, or common periodontal procedures and treatments. In addition, DESS-OPGs or SPACE-STIR OPGs (with a scan time of 12 min) can potentially lead to the early detection of various anatomic abnormalities or potentially unexpected pathologies that cannot be identified by conventional OPGs with little time and effort. This, in turn, may result in improved patient outcomes.

### Limitations

Several limitations should be acknowledged: First, the sample size of 21 patients does not allow for extrapolations of generally valid conclusions and should be considered a methodological limitation. Larger cohorts and further studies are required to obtain further insights and knowledge of MR-OPGs. Second, the included study participants were relatively young, leading to the need for age-matched controls to assess artifacts caused by dental restorations and implants as they increase with age. Third, fixed orthodontic retainers are a significant source of artifacts in the anterior teeth region^40^, making it impossible to assess the incisor teeth. This is a remaining concern in dental MRI that requires further refinement of the MRI sequences to minimize this type of artifacts. However, the occurrence of artifacts caused by dental restorations, dental implants, and orthodontic appliances remains a major concern for all sequences except UTE, which exhibits no or minor artifacts.

## Conclusion

MR-OPGs are a promising advance in diagnostic imaging for operative dentistry and oral and maxillofacial surgery that provides high spatial resolution images of hard and soft tissues in the oral cavity without the use of ionizing radiation. They represent another step toward personalized medicine, enabling accurate depiction of anatomy, detection of pathologies, staging, and radiological follow-up of oral and maxillofacial diseases, providing advantageous information compared to conventional imaging modalities. MR-OPGs could initiate a paradigm shift in oral and maxillofacial radiology by enabling case- and indication-specific perioperative imaging instead of radiation-based standardized OPGs, that will potentially minimize risks and complications during surgical procedures.

## Data Availability

The datasets used during and/or analysed during the current study are available from the corresponding author on request.
