# Author Correction: MR-orthopantomography in operative dentistry and oral and maxillofacial surgery: a proof of concept study

**DOI:** 10.1038/s41598-023-34473-5

**Published:** 2023-05-08

**Authors:** Adib Al-Haj Husain, Valérie Schmidt, Silvio Valdec, Bernd Stadlinger, Sebastian Winklhofer, Daphne Schönegg, Stefan Sommer, Mutlu Özcan, Nadin Al-Haj Husain, Marco Piccirelli

**Affiliations:** 1 Clinic of Cranio-Maxillofacial and Oral Surgery, Center of Dental Medicine, University of Zurich, Plattenstrasse 11, CH-8032 Zurich, Switzerland; 2 Department of Neuroradiology, Clinical Neuroscience Center, University Hospital Zurich, University of Zurich, Zurich, Switzerland; 3 Departement of Cranio-Maxillo-Facial and Oral Surgery, University Hospital Zurich, Zurich, Switzerland; 4 Siemens Healthineers International AG, Zurich, Switzerland; 5 Swiss Center for Musculoskeletal Imaging (SCMI), Balgrist Campus, Zurich, Switzerland; 6 Advanced Clinical Imaging Technology (ACIT), Siemens Healthcare AG, Lausanne, Switzerland; 7 Division of Dental Biomaterials, Clinic of Reconstructive Dentistry, Center of Dental Medicine, University of Zurich, Zurich, Switzerland; 8 Departement of Reconstructive Dentistry and Gerodontology, School of Dental Medicine, University of Bern, Bern, Switzerland

Correction to: *Scientific Reports* 10.1038/s41598-023-33483-7, published online 17 April 2023

The original version of this Article contained an error in the Materials and methods section.

In the Materials and methods section, under the subheading ‘Study design’,

“This cohort study was conducted in collaboration between the Department of Neuroradiology *(Department of Neuroradiology of the University Hospital Zurich and the Clinic of Cranio-Maxillofacial and Oral Surgery of the University of Zurich)* and the Clinic of Cranio-Maxillofacial and Oral Surgery of the *(Clinic of Cranio-Maxillofacial and Oral Surgery of the University of Zurich)* and included 21 volunteers.”

now reads:

“This cohort study was conducted in collaboration between the Department of Neuroradiology of the University of Zurich and the Clinic of Cranio-Maxillofacial and Oral Surgery of the University of Zurich and included 21 volunteers.”

The original Article has been corrected.

